# Coenzyme Q_10_: Novel Formulations and Medical Trends

**DOI:** 10.3390/ijms21228432

**Published:** 2020-11-10

**Authors:** Carmen J. Pastor-Maldonado, Juan M. Suárez-Rivero, Suleva Povea-Cabello, Mónica Álvarez-Córdoba, Irene Villalón-García, Manuel Munuera-Cabeza, Alejandra Suárez-Carrillo, Marta Talaverón-Rey, José A. Sánchez-Alcázar

**Affiliations:** Centro Andaluz de Biología del Desarrollo (CABD-CSIC-Universidad Pablo de Olavide), and Centro de Investigación Biomédica en Red: Enfermedades Raras, Instituto de Salud Carlos III. Universidad Pablo de Olavide, 41013 Sevilla, Spain; carmenj3b@gmail.com (C.J.P.-M.); juasuariv@gmail.com (J.M.S.-R.); sulevapovea@gmail.com (S.P.-C.); monikalvarez11@hotmail.com (M.Á.-C.); villalon.irene@gmail.com (I.V.-G.); mmuncab@upo.es (M.M.-C.); asuacar1@gmail.com (A.S.-C.); martatalrey@gmail.com (M.T.-R.)

**Keywords:** Coenzyme Q_10_, ubiquinone, mitochondria

## Abstract

The aim of this review is to shed light over the most recent advances in Coenzyme Q_10_ (CoQ_10_) applications as well as to provide detailed information about the functions of this versatile molecule, which have proven to be of great interest in the medical field. Traditionally, CoQ_10_ clinical use was based on its antioxidant properties; however, a wide range of highly interesting alternative functions have recently been discovered. In this line, CoQ_10_ has shown pain-alleviating properties in fibromyalgia patients, a membrane-stabilizing function, immune system enhancing ability, or a fundamental role for insulin sensitivity, apart from potentially beneficial properties for familial hypercholesterolemia patients. In brief, it shows a remarkable amount of functions in addition to those yet to be discovered. Despite its multiple therapeutic applications, CoQ_10_ is not commonly prescribed as a drug because of its low oral bioavailability, which compromises its efficacy. Hence, several formulations have been developed to face such inconvenience. These were initially designed as lipid nanoparticles for CoQ_10_ encapsulation and distribution through biological membranes and eventually evolved towards chemical modifications of the molecule to decrease its hydrophobicity. Some of the most promising formulations will also be discussed in this review.

## 1. Introduction

Coenzyme Q_10_ (CoQ_10_) was first identified back in the fifties by two groups, Festenstein et al. (1955) [1] and Crane et al. (1957) [2]. Its name was chosen due to the fact that it is an ubiquitous quinone present in all cells and that its chemical structure comprises a quinone group with a variable number of isoprenyl units, being ten in the case of humans [3]. Its reduced form is known as ubiquinol and the oxidized one as ubiquinone. Both forms coexist and through sequential redox reactions serve to regenerate each other (Q cycle) [4]. The biological relevance of this vital molecule is related to its functions as a lipophilic antioxidant (scavenges free radicals inhibiting the initiation and progression of lipid peroxidation in cell membranes) [5]. Moreover, the intracellularly produced ubiquinone is a potent endogenous antioxidant and acts as an electron carrier in the mitochondrial electron transport chain, being involved in electron transport between complexes I or II and complex III [6]. Ubiquinol, meanwhile, presents antioxidant behavior in cell and organelle membranes. Apart from these main functions, CoQ_10_ plays an important role in uncoupling proteins’ activation [7], cell signaling [8], cell growth [9] and apoptosis through the modulation of the mitochondrial permeability transition pore [10]. This molecule has traditionally been used clinically as an enhancer of mitochondrial function or an antioxidant intended to either palliate or diminish the oxidative damage that may worsen the physiological outcome of a wide range of diseases. For this purpose, it is commonly administered orally as a dietary supplement. However, this may compromise the effectivity of treatment, since the intestinal absorption of the molecule is poor. In fact, studies in rats demonstrate that only about 3% of the orally administered CoQ_10_ can be absorbed [11]. In this case, increasing the dosage is not a feasible solution due to the toxicity associated with the traditional vehicles used for its delivery at high concentrations. The acceptable daily intake (ADI) of CoQ_10_ has been set at 12 mg/kg/day. This is calculated from the no-observed-adverse-effect level (NOAEL) by applying a safety factor of 100 (i.e., ≥600 mg/day for adults weighing 50 kg) [12].

CoQ_10_ is indispensable for the proper functioning of any cell type, hence, its absence or its reduced synthesis has severe consequences. The diverse phenotypes associated with low CoQ_10_ concentration are provoked by an abnormal respiratory chain function in addition to increased generation of free radicals, leading to inadequate cellular energy production and consequent degradation of mitochondria by mitophagy [13]. The repercussions of these failures are various diseases that range from cerebellar ataxia, severe infantile multisystemic failure, and encephalomyopathy or nephropathy to isolated myopathy or nephrotic syndrome with or without sensorineural hearing loss, these latter being the most common ones [14].

**Figure 1:**
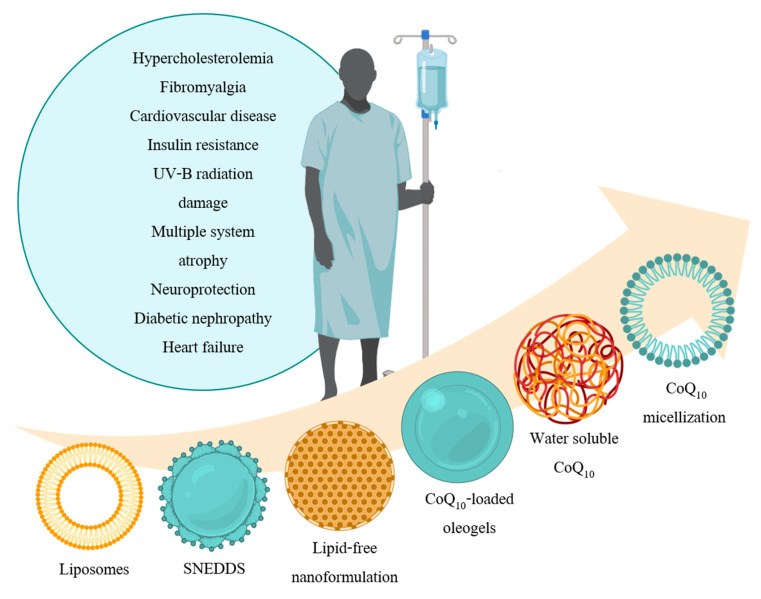
CoQ_10_ applications in the medical field. Traditionally, CoQ_10_ has been used as a therapeutic agent for diabetic cardiovascular diseases, diabetic nephropathy, neuroprotection, and heart failure owing to its antioxidant activity. In fact, it is widely known that the excessive generation of ROS is a common underlying factor of all these diseases which severely worsens the outcome for the patients. More recently, several studies have proved CoQ_10_’s potential application against ultraviolet B (UVB) radiation damage, multiple system atrophy (MSA), or as an immunity and inflammatory marker. Both in MSA and inflammation, CoQ_10_ levels are below the normal range in patients, the reason why its supplementation led to an improvement in their development and well-being. Furthermore, due to its ROS-scavenging function, CoQ_10_ has proved to be significantly beneficial for skin cells’ protection against UVB radiation and insulin sensitivity. Surprising though it may seem, it is also highly beneficial for familial hypercholesterolemia patients who, in general, present a secondary CoQ_10_ deficiency derived from their disease. Finally, it has shown a remarkable membrane stabilizing property, since it increases the lipid packing order and the mechanical stability of the IMM-mimicking membranes, the reason why it could potentially be used as a therapy for Barth syndrome patients.

**Figure 2:**
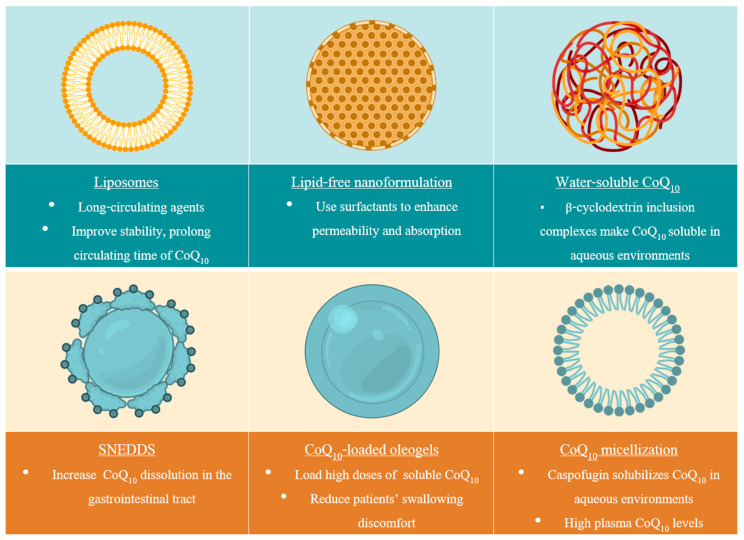
Highlights of CoQ_10_ formulations. The initial trend in CoQ_10_ formulations’ design was to use non-polar agents as delivery systems due to several advantages they present (increase drug solubility in intestines, recruit lymphatic drug transport, or modify enterocyte-based drug transport and disposition). However, there is rising interest in the development of water-soluble delivery mechanisms in order to prevent the common drawbacks of lipid-based formulations, such as rate of dispersion, degree of emulsification, particle size, or drug precipitation from the formulation upon dispersion. The first attempts towards this goal include the development of lipid-free CoQ_10_ nanoformulations, micellar CoQ_10_ solutions, and hydrophilic CoQ_10_ inclusion complexes.

In the present review, the most recent CoQ_10_ applications of therapeutic and scientific interest will be examined (Figure 1 and Table 1), as well as the state-of-the-art CoQ_10_ formulations (Figure 2). To conclude, future perspectives associated with the use of this versatile molecule will be discussed to give a general overview of the promising advances that the field can undergo.

Coenzyme Q_10_ has been extensively used in the clinic due to its potent antioxidant properties. However, less known CoQ_10_ properties have gained importance in the medical field in the past few years. Now that the physiological barriers of CoQ_10_ delivery have virtually been overcome, this quinone could prove an invaluable ally for the treatment of a wide spectrum of diseases. This table lists some of the most recent clinical applications of CoQ_10_ and the diseases that would benefit from them.

## 2. CoQ_10_ Applications in the Medical Field

### 2.1. Diabetic Cardiovascular Diseases and Diabetic Nephropathy

Diabetes mellitus is a metabolic disorder characterized by impaired insulin production and reduced insulin sensitivity. This disorder leads to inadequate glucose storage and deficient glycogenolysis and gluconeogenesis pathways. As a result, patients suffer from chronic hyperglycemia and, subsequently, from long-term micro- and macrovascular complications. The microvascular complications affect small blood vessels and include nephropathy, neuropathy, and retinopathy, while the macrovascular complications comprise cardiovascular, cerebrovascular, and peripheral artery diseases [15].

Macrovascular complications of diabetes, such as diabetic cardiovascular disease (CVD), occur through a number of hyperglycemia-induced mechanisms that trigger generation of oxidative stress. The generation of reactive oxygen species (ROS) by diabetic cardiac cells can be mediated by several pathways, such as, among other things, activation of the receptor for advanced glycation end products (RAGE) by advanced glycation end products (AGEs), xanthine oxidase, nicotinamide adenine dinucleotide phosphate (NADPH) oxidase, activation of protein kinase C (PKC) [11]. Given that these factors interact, profound overproduction of ROS takes place, leading to diabetic CVD. The symptoms of this lateral disease include, among other things, alterations in cardiac morphology, increased blood pressure, and chest pain [16].

Diverse antioxidant molecules have been tested for possible therapeutic use. Among them, CoQ_10_ has shown promising results. A recent randomized controlled trial has proven that its use as an adjunct therapy for chronic heart failure is associated with a significant reduction in adverse cardiovascular events [17]. For this reason, CoQ_10_ can be considered a potential treatment for CVD patients.

Diabetic nephropathy (DN) is one of the most recurrent microvascular complications associated with hyperglycemia. A recent approach towards this disease’s treatment involves the combination of CoQ_10_-loaded liposomes with ultrasound-targeted microbubble destruction (UTMD) [18]. As previously mentioned, excessive ROS are found in the mitochondrial respiratory chain in high-glucose environments [19]. High ROS concentrations are the most advanced and upstream factor in the pathogenesis of diabetic vascular disease [20] and hence of diabetic nephropathy. Thereby, CoQ_10_ with its antioxidant function could be a potential treatment for early DN. Nevertheless, CoQ_10_ has limited clinical application owing to its low bioavailability. In order to resolve this problem, liposome-mediated delivery systems might be the best option, not only because they enable the transport of CoQ_10_ to the target tissue, but also because they achieve a successful release of the cargo, reduce drug toxicity, and improve drug stability thanks to their good cell compatibility [21]. Yet, CoQ_10_ delivery would be inefficient and non-specific. Aiming to ameliorate the delivery system, liposomes are combined with UTMD. Previous studies confirm that this combination increases local renal interstitial capillary permeability [22], facilitating the entrance of CoQ_10_ in the tissue. This delivery method proved highly satisfactory to treat impaired kidney. The authors of the study believe that the success of the technique can be attributed to the use of UTMD, since it impelled the CoQ_10_-charged liposomes to the target kidney in a non-invasive fashion [17].

### 2.2. Multiple System Atrophy

Multiple System Atrophy (MSA) is an incurable and progressive neurodegenerative condition. Clinically, it is grouped with atypical parkinsonism since its main symptoms are parkinsonism or ataxia plus dysautonomia [23]. Up to the moment, the diagnose of such disease could only be confirmed neuropathologically, being misdiagnoses relatively common [24,25]. Recent studies have proposed low CoQ_10_ levels in cerebrospinal fluid (CSF) as an accurate biomarker for MSA since mutations on the coenzyme Q2 (*COQ2*) gene and an abnormally low cerebellar CoQ_10_ concentration have been reported in all MSA patients. The overlap in the levels of CoQ_10_ in plasma and serum [26,27] and the dependence on other modifiers or confounders like cholesterol [20], were the key aspects leading researchers to hypothesize CSF as an alternative and reliable source to assess a possible MSA biomarker [28]. Interestingly, there is a significant lowering of CoQ_10_ levels in CSF from MSA patients in comparison with other Parkinsonism sufferers and healthy individuals; irrespective of the presence of other confounders [22]. In this line, there is available evidence that CoQ_10_ deficiency is more specifically related to MSA than to other neurodegenerative diseases [29]. Therefore, ubiquinol, a reduced form of CoQ_10_, has been used to treat patients suffering from MSA due to COQ2 mutations with satisfactory results [30].

### 2.3. Immunity and Immunological Disorders

The clinical outcomes of CoQ_10_ deficiency are variable depending on the genetic heterogenicity involving either CoQ_10_ biosynthetic pathways (primary defect) or other pathways (secondary defect) [31]. Recent studies have related impaired immune function with CoQ_10_ deficiency in a case report [14]. The patient showed no mutations of the genes known to be linked to primary or secondary CoQ_10_ deficiency (*COQ2*, *COQ9*, *COQ8A*, *ETFDH*, and *PDSS2*), however, she presented extremely low levels of muscular CoQ_10_ (0.02 µg/mg in comparison to the normal levels of 0.14–0.29 µg/mg protein). Additionally, abnormal T cell proliferation was reported, together with a decreased fraction of plasmablasts, which contributed to the poor humoral response noted in the patient [14]. Proliferating immune cells have a high energy demand [32]. Leukocytes, in particular, are rich in CoQ_10_ and require a reliable mitochondrial network [33]. Thereby, CoQ_10_ supplementation was administered, with the results being highly satisfactory, since a significant improvement of the T cell function was detected. Moreover, the patient was subject to an immunoglobulin replacement treatment which, combined with CoQ_10_ supply, resulted in a reduced number of infections, less hospitalizations, and, overall, a better quality of life.

It would be convenient to carry out further investigation on the impact of CoQ_10_ deficiency over immune function so as to elucidate whether functional T cell deficiency may be linked to CoQ_10_ deficiency in patients with recurrent infections. This would explain the improvement of the CD4 T cell counts in acquired immune deficiency syndrome (AIDS) patients following a CoQ_10_ supplementation treatment [34,35].

Surprising though it may seem, CoQ_10_ supplementation has also been proven to reduce the levels of circulating inflammatory markers [36]. According to previous studies, inflammation plays a deleterious role in numerous diseases, such as, among other diseases, type 2 diabetes [37], CVD [38], chronic obstructive pulmonary disease [39], and cancer [40]. Elevated levels of inflammatory markers, such as C-reactive protein (CRP), interleukin 6 (IL-6), and tumor necrosis factor α (TNF-α), increase the risk of chronic disease development and contribute to enhance disease pathogenesis [41]. Oxidative stress is one of the main factors that trigger inflammation [42], therefore, antioxidants that target oxidative stress and inflammation like CoQ_10_ may be highly effective in disease prevention or treatment [30,43]. The anti-inflammatory effect of CoQ_10_ is already widely known [42], nonetheless, the most recent approaches are aimed at examining the impact of CoQ_10_ over inflammatory mediators CRP, IL-6, and TNF-α. The ability of CoQ_10_’s to lower these inflammatory mediators was reported in a systematic review and meta-analysis carried out in the past few years [30]. This is deeply interesting since the data show that the accumulation of such mediators has detrimental consequences on our health. As a matter of fact, elevated CRP, product of hepatic stimulation under IL-6 regulation, is a notable risk for CVD and diabetes [44,45]. Furthermore, IL-6 and TNF-α are closely related to chronic kidney disease [46], type 2 diabetes [47], and CVD [48]. Presumably, CoQ_10_ anti-inflammatory potential is related to the downregulation of nuclear factor-ϰβ (NF-ϰβ)-dependent gene expression [49], which is known to be activated by reactive oxygen species [50]. With NF-ϰβ inhibited, no expression of pro-inflammatory cytokines could take place. Bearing this in mind, further studies should be encouraged to translate these findings into clinical applications for the improvement of patients’ well-being.

### 2.4. Neuroprotection

Neurodegeneration, the progressive loss of neuronal function, electrical activity, and structure in the central nervous system (CNS) is the main triggering feature of acute and chronic neurodegenerative diseases, such as, among other diseases, Alzheimer’s disease, Parkinson’s disease, paraneoplastic disorders, or multiple sclerosis [51]. The most common neurodegeneration-promoting factors are neuroinflammation, oxidative stress, mitochondrial dysfunction, protein aggregation and misfolding, excitotoxicity, cell death pathways, and the loss of trophic factors [52].

Oxidative stress results from the accumulation of free radicals due to either the overproduction of ROS or the failure of cellular buffering mechanisms. In line with this, it has been reported that the CNS cells of most Parkinson’s disease (PD) patients show high oxidative damage in their proteins, nucleic acid, or lipids [53]. The excessive production of ROS occurs as a consequence of either dopamine metabolism [54], mitochondrial dysfunction mostly caused by impaired complex I in the nervous system of PD sufferers [55], or environmental factors (exposure to ultraviolet radiation).

For instance, the neuronal ganglion cells of the retina are highly exposed to sun radiation, being therefore subject to acute oxidative stress [56]. Hence, CoQ_10_ with its antioxidant properties is a crucial molecule for their protection against the oxidative damage perpetrated by ROS [57]. Nonetheless, concentrations of CoQ_10_ in the human retina are reported to decline with age [58], meaning that an external supplementation of the molecule is necessary to maintain optical health as a person ages. Topically active formulations of CoQ_10_ have successfully been developed [59,60], but their efficacy is compromised by poor aqueous solubility of this molecule and its low bioavailability. It is widely known that the multi-drug efflux pump P-glycoprotein (P-gp) has a lot to do with this limited bioavailability [61]. Ergo, the novel approaches aimed at enhancing the topical delivery and the pharmacological effects of CoQ_10_ consider the administration of this drug together with a P-gp inhibitor [62]. One of the tested inhibitors has been α-tocopherol polyethylene glycol 1000 succinate (TPGS), which formulated into micelles with CoQ_10_, successfully increased its delivery [63]. Recent studies have gone deeper in the subject by trying to demonstrate the neuroprotective activity of CoQ_10_/TPGS compared to CoQ_10_ alone. According to their results, CoQ_10_/TPGS micelles are significantly more neuroprotective against retinal ganglion cell (RGC) loss [61]. This is in agreement with the previous work in the field which proved that co-administration of CoQ_10_ with an α-tocopherol derivative enhances the neuroprotective activity of CoQ_10_ in vitro [64,65]. The postulated mechanisms through which CoQ_10_ is thought to elicit its neuroprotective function are its antioxidant activity [66] and its Ca^2+^ buffering activity [67], due to the fact that accumulation of intracellular Ca^2+^ is known to signal for apoptosis induction [68].

Another fundamental application of Coenzyme Q_10_ in the field of ocular health is the therapeutic potential of this molecule to treat glaucoma. Glaucoma is the leading cause of irreversible blindness worldwide [69]. There is compelling evidence to suggest that apart from optic nerve degeneration and retinal ganglion cell death, glutamate excitotoxicity and oxidative stress are key pathophysiological mechanisms in mitochondrial dysfunction-mediated glaucomatous neurodegeneration [70,71]. CoQ_10_, with its ability to maintain the mitochondrial membrane potential while supporting ATP synthesis and inhibiting reactive oxygen species’ generation, has proven to be an invaluable therapeutic tool for the protection of neuronal cells against oxidative stress in neurodegenerative conditions like glaucoma. Indeed, intraocular CoQ_10_ administration had a potent neuroprotective effect on the established animal models of retinal ischemia [72]. According to the authors of the study, it prevented high intraocular pressure-derived optic nerve degeneration and RGC death [73]. Moreover, recent approaches have proven that dietary supplementation with CoQ_10_ not only promotes RGC survival in DBA/2J mice, but also reduces the expression of proapoptotic protein Bax in the retina of glaucomatous mice. Based on the available evidence, it is now clear that CoQ_10_ prevents retinal ganglion cell death by diminishing oxidative stress and glutamate excitotoxicity and by inhibiting the activation of the Bax-mediated apoptotic pathway [74]. In light of the success of Coenzyme Q_10_ supplementation to treat glaucoma in mice, it would be reasonable to expect its incorporation to the clinic to be nothing but a matter of time.

Patients with vascular disorders affecting the retina also benefit from CoQ_10_ supplementation, as proven in a retrospective clinical case study with 48 patients. The interruption of CoQ_10_ treatment resulted in a pronounced decrease of the patients’ visual field index, which was recovered once the treatment was restored [75].

Neuroinflammation has been identified as a primary mechanism involved in neurodegenerative diseases’ pathogenesis. In this regard, activation of microglia has been detected in the nervous system and striatum from postmortem PD patients’ brains. Moreover, pro-inflammatory cytokines’ levels have been reported over the mean values in the CSF and basal ganglia of neurodegenerative diseases’ patients [52]. Active microglia cells release cytokines, including interleukin 1β (IL-1β) and TNF-α, which promote inflammation. Thereby, microglia inhibitory compounds or anti-inflammatory substances represent promising neuroprotective strategies [76].

Several diseases have been related to abnormal microglial activation (microgliosis) [42]. One of them is epilepsy, whose pathophysiology is characterized by both a severe mitochondrial dysfunction and neuroinflammation [77]. CoQ_10_ with its ability to downregulate nuclear factor-ϰβ (NF-ϰβ)-dependent gene expression prevents production of pro-inflammatory cytokines [35]. Furthermore, recent studies [78] have demonstrated the effectivity of CoQ_10_ as a treatment for epilepsy due to its interaction with minocycline, a second generation tetracycline which suppresses microglial activation and reduces TNF-α release, being beneficial to treat various neurological problems [79].

The outcome of the combined treatment of patients with both CoQ_10_ and minocycline has revealed the synergistic effect of both molecules and their potential as a cocktail against cognitive dysfunction-related diseases [53]. These findings also suggest that they might share overlapping mechanisms of action, a topic which deserves further in-depth research.

### 2.5. Against UVB Radiation Damage

UVB radiation triggers major alterations in skin cells, with ROS overproduction being one of the most deleterious. In fact, several pathologic changes in the skin, such as erythema, edema, sunburn, immune suppression, or cancer occur as a consequence of the excessive production of reactive oxygen species [80,81]. The most common and widespread protective method against ultraviolet (UV) radiation damage is the topical application of regular sunscreens, which absorb, reflect, or scatter radiation. However, they are not capable of repairing cutaneous injuries. For this reason, there is a growing search for antioxidant molecules’ formulations that can alleviate cytotoxic events derived from ROS intracellular accumulation [82]. CoQ_10_ and vitamin E are both known to be highly antioxidant molecules that play fundamental roles in most biological systems. Hence, their combination was hypothesized as a promising strategy to boost their cytoprotective potential [83]. The challenge that researchers had to face in this regard was the implementation of a viable delivery system. In the past few years, nanocapsules have emerged as promising mechanisms for topical substance delivery due to their ability to control drug release and improve drug stability [84]. Thereafter, the newest approaches towards an effective skin repair treatment have been based on the formulation of CoQ_10_ and vitamin E nanocapsules. In the process, the novel association between CoQ_10_ and vitamin E nanocapsules via gellan gum, an amicrobial polysaccharide with gelling properties [85], was demonstrated [66]. The relevance of this study lies in the formulation of a semisolid nanoparticle system thanks to the addition of gellan gum. This is interesting due to the fact that a semisolid texture would enable the direct cutaneous application of the drug-containing nanoparticles. Furthermore, the anti-edematogenic, anti-inflammatory, and antioxidant activities of these semisolid formulations were tested in an animal model of UVB radiation inflammation and yielded promising results [66].

### 2.6. For Heart Failure Treatment

Apart from its antioxidant and electron carrier functions, CoQ_10_ displays a wide range of interesting properties for the treatment of cardiovascular diseases: its positive influence on myocardial Na^+^–K^+^ ATPase activity, its protective effect on endothelial cells, its influence on prostaglandin metabolism, and its anti-viscosity effect [86,87]. Concretely, chronic heart failure patients enormously benefit from CoQ_10_ supplementation for the amelioration of their condition. This comes down to the fact that CoQ_10_ levels were found to be significantly decreased in blood and endomyocardial biopsies of heart failure patients. Moreover, such a decrease correlated with severity of the symptoms, the patients whose CoQ_10_ levels were lower being more affected. Based on this finding, the effect of CoQ_10_ supplementation was tested on a group of cardiovascular disease patients. The results backed up the premise of CoQ_10_ effectivity, being beneficial for 69% and 43% of patients with cardiomyopathy and ischemic heart disease, respectively [88]. In order to face heart failure, CoQ_10_ supplementation must be high enough to increase plasma CoQ_10_ concentration to therapeutic levels (>2.5 μg/mL). Studies confirm that 300 mg/day of orally administered CoQ_10_ increases plasma CoQ_10_ concentration to 3.25 ± 1.5 μg/mL [89,90]. The diversity of its beneficial effects as well as the satisfactory results obtained in previous studies set CoQ_10_ as the first treatment to improve survival in heart failure patients ahead of angiotensin-converting enzyme (ACE) inhibitors and β-blockers more than a decade ago [91].

Furthermore, plasma CoQ_10_ levels have been proposed as a reliable biomarker to predict mortality in chronic heart failure (CHF). A recent study with a cohort of 236 chronic heart failure patients demonstrated that reduction in CoQ_10_ levels strongly correlates with worse outcome in CHF [92].

Another interesting CoQ_10_ application for the cardiology field was recently discovered. Several studies claim that CoQ_10_ can be used as a marker of rejection in patients who underwent heart transplantation. Low CoQ_10_ concentrations were observed in the plasma and endomyocardial biopsies of patients who presented signs of histological rejection, whilst patients without a rejection reaction showed normal CoQ_10_ levels [82]. Furthermore, it was postulated that CoQ_10_ supplementation could be conceived as an option to prevent transplant rejection [93].

### 2.7. Barth Syndrome and Membrane Instability-Related Diseases

Barth syndrome is a cardiomyopathy that includes skeletal muscle weakness, neutropenia, and growth retardation [94]. The locus associated with this disease has recently been mapped to the *Xq28* (tafazzin) gene. The consequence of this gene’s mutation is an impairment of the phospholipid metabolism that results in a profound cardiolipin deficiency in patients [95]. Cardiolipin is a dimeric phospholipid that is almost exclusively found in the inner mitochondrial membrane (IMM) of mammalian cells [96]. Its functional relevance arises from its unique ability to interact with proteins and its role in maintaining IMM stability and fluidity [97]. Absence or depletion of cardiolipin translates into mitochondrial dysfunction as documented in a variety of pathological conditions: hypo/hyperthyroidism [98,99], ischemia [100], heart failure [101], aging [102], and Barth syndrome and other related diseases associated with mitochondrial impairment. Reconstituting cardiolipin levels in patients has therefore been the aim of several therapeutic strategies developed against these disorders. One of the most successful approaches proposed the exogenous supplementation of cardiolipin liposomes to restore mitochondrial cytochrome bc1 (complex III) functionality [103]. However, there is an increasing availability of therapeutic options for mitochondrial dysfunction beyond cardiolipin. For instance, detailed studies on both the biological and physiological roles of CoQ_10_ have revealed a mechanical stability enhancing function of this molecule on cell membranes. Among them, a study on the impact of CoQ_10_ on membrane density, permeability, resistance to solubilization by detergents, and promptness to rupture of 1-palmitoyl-2-oleoyl-sn-glycero-3-phosphocholine (POPC) liposomes, liposomes formed by pure lipids, and liposomes supplemented with cholesterol or solanesol is worth pointing out. The results obtained support the hypothesis of a stabilizing role of CoQ_10_ in phospholipid membranes, since POPC nanoparticles became more resistant to rupture and less permeable to hydrophilic solutes in its presence. From the fact that solanesol (a polyisoprenoid alcohol structurally similar to CoQ_10_ with 9 isoprene units rather than 10) [104] had no effect on the analyzed membrane properties, it was elucidated that the quinone moiety is important for the stabilizing function of CoQ_10_. This discovery is of great interest since it implies that CoQ_10_ could be used as a membrane-stabilizing agent for the treatment of such diseases as Barth syndrome, where the lack of cardiolipin compromises the structural integrity and functionality of the mitochondrial membrane.

Moreover, this study reported that the condensing effect of CoQ_10_ was qualitatively similar to that of cholesterol, with a significantly smaller amount of CoQ_10_ needed to produce the same effect on, e.g., lipid packing. In line with this, cholesterol could be substituted by CoQ_10_ for various purposes, for example, the development of cholesterol-free liposome-based drug delivery systems [105].

It must be stressed that the former study was carried out on POPC liposomal membranes, whereas native biological membranes contain, as a rule, a complex mixture of different lipid species. Such alterations in the lipid composition can lead to relevant changes in lipid spontaneous curvature, membrane binding rigidity, and other properties that could affect CoQ_10_’s interaction with or effect on the membrane. In fact, it has been proven that lipid CoQ_10_ interaction depends very much on the lipid species [105]. Nonetheless, the effects observed in the POPC membranes persisted in an alternative research project which made use of membranes with a lipid composition resembling that of the IMM. In fact, it was observed that CoQ_10_ not only increased the lipid packing order and the mechanical stability of the IMM-mimicking membranes, but also improved their barrier properties, which implies that it has a stabilizing role in biological membranes [106].

In this line, the role of acyl chains or intermembrane proteins on membrane stability should be further studied in order to have a better understanding of CoQ_10_’s stabilizing function of the IMM. It must be pointed out that the protein load in the IMM is high. This could be a prominent interfering factor in future experimental approaches, since high protein content is known to lead to lipid segregation and other processes that impact overall membrane structure and dynamics [106].

### 2.8. Insulin Resistance

Mitochondrial oxidants have been reported to play a fundamental role in the development of insulin resistance in adipose [107] and muscle tissue [108]. Supporting this observation, mass spectrometry-based proteome analysis of adipose tissue from insulin-resistant 3T3-L1 mouse adipocytes and adipose tissue from insulin-resistant human cells has revealed downregulation of the mevalonate/CoQ_10_ biosynthetic pathways in both systems. This abnormality eventually leads to a decrease in the mitochondrial CoQ_10_ content. The rationale behind this is that triggering insulin resistance promotes hyperinsulinemia, inflammation, corticosteroids, or caloric excess and prevents the expression of CoQ_10_ biosynthetic enzymes and, hence, lower CoQ_10_ synthesis rates. As a consequence of the reduced CoQ_10_ biosynthesis, the levels of superoxide rise notoriously in cells as proven by highly specific mass spectrometry-based superoxide assays [109]. The majority of the superoxide in mitochondria is derived from flavin sites, including that in complex II (IIF) [110]. Therefore, it is hypothesized that excessive superoxide production from the IIF site occurs as a consequence of increased concentrations of the flavin radical, which is overproduced as a result of scarce electron transfer to the non-abundant CoQ_10_ [109]. It is widely known that scavenging mitochondrial oxidants benefits insulin sensitivity [107,108]. Nevertheless, the mechanism through which oxidants impair insulin action remains uncertain. According to recent studies, it is highly likely that there is a presently undiscovered signal transduction pathway that directly communicates mitochondria and oxidants to the mediators of insulin action in the cytoplasm [109].

For the reasons that have previously been discussed, CoQ_10_ has received considerable attention as a supplement to treat patients suffering from diabetes. However, low oral bioavailability of CoQ_10_ represents a substantial limitation, especially in situations of CoQ_10_ deficiency, where mitochondrial homeostasis needs to be restored in metabolic tissues [111]. Therefore, a pharmaceutical alternative to supplementation would be to target cellular processes that play a part in the regulation of the mitochondrial CoQ_10_ content. Further therapeutic approaches have been proposed, such as targeting complex II as the site of increased superoxide production in response to the loss of mitochondrial CoQ_10_. Up to now, several compounds that prevent oxidant production from complex I [112] and III [113] have been identified. The identification of a similar substance for complex II would be an essential breakthrough to mitigate superoxide production in patients and thus to overcome insulin resistance [109].

Finally, it has been proven that mevalonate pathway-inhibiting statins lower the CoQ_10_ content and trigger insulin resistance in a CoQ_10_-dependent manner. This might shed light on the relationship between statin therapy in humans and insulin resistance [109,114,115].

### 2.9. Pain Alleviation in Fibromyalgia

Fibromyalgia (FM) is a chronic pain syndrome whose pathophysiological mechanisms have not yet been identified. Extensive research in the field has unraveled a direct link between oxidative stress and the pathogenesis of such a disease [116] being therefore mitochondrial dysfunction proposed as one of the main triggering factors of fibromyalgia. In fact, a decrease in mitochondrial mass and CoQ_10_ levels, as well as an overproduction of ROS, was detected in blood mononuclear cells from FM patients [117]. It was also reported that these cells actively remove damaged mitochondria via mitophagy [117] but cannot rely on the normal mitochondrial compensatory mechanisms, since they are impaired. As a consequence, they have a reduced mitochondrial mass and a depleted amount of antioxidant molecules [118], which makes them prone to inflammatory damage. Accordingly, these cells present enhanced activation of the inflammasome owing to CoQ_10_ deficiency, as well as increased serum levels of proinflammatory cytokines, such as IL-1β and interleukin 18 (IL-18) [119]. As it is observed in major metabolic diseases, such as diabetes and insulin resistance, the activation of the inflammasome is a sign of metabolic danger and stress [120].

Interestingly, inflammation has been associated with FM symptoms by means of high positive correlations between IL-1β and IL-18 serum levels and pain scores. This suggests that there is an inflammatory component in pain induction. It has also been proven that inflammation in FM is dependent on mitochondrial dysfunction, since: (1) a negative correlation has been found between proinflammatory cytokines (IL-1β and IL-18) and CoQ_10_ and (2) a positive one has been found between these cytokines, mitochondrial ROS, and pain scale scores [119]. In brief, the involvement of CoQ_10_ deficiency in the pathological process of inflammasome activation and release of proinflammatory cytokines has been revealed [121]. For this reason, CoQ_10_ presents a potential treatment for pain alleviation in FM patients. Still, further clinical studies should be carried out to corroborate the effectiveness of this antioxidant therapy in such a pathology.

### 2.10. Familial Hypercholesterolemia and Atherosclerosis

By definition, hypercholesterolemia is a physiological condition at which an abnormally high amount of cholesterol is present in the blood of a patient. This condition can either be triggered by hereditary mutations (familial hypercholesterolemia) on the genes involved in the low-density lipoprotein (LDL) clearance mechanism or by a non-healthy lifestyle, which is especially predominant among the elderly [122]. According to a recent study, patient-derived familial hypercholesterolemia (FH) fibroblasts bearing a mutation on the LDL receptor (LDL-R) are unable to import cholesterol for its metabolism. Since cholesterol uptake is impaired, these fibroblasts synthesize such lipid endogenously in an uncontrolled manner, so that it eventually accumulates within them [123]. The upregulation of cholesterol biosynthesis is directly related to the 3-hydroxy-3-methyl-glutaryl-coenzyme A reductase (HMGCR) activity, which is itself controlled by lipoproteins binding to the LDL-R [124]. Bearing in mind that these FH fibroblasts have dysfunctional LDL-R or lack them, it is not surprising that such a cholesterogenic enzyme is upregulated. As a consequence, the mevalonate pathway shifts to overproduction of cholesterol in detriment of CoQ_10_ biosynthesis, meaning that these FH patients suffer from secondary CoQ_10_ deficiency. The lack of CoQ_10_ together with cholesterol accumulation is associated with mitochondrial function impairment, which is characterized by an elevated ROS production, reduced ATP levels, low activity of the mitochondrial respiratory complexes, and mitochondrial depolarization. Indeed, mitochondrial dysfunction in these FH fibroblasts with mutations on the LDL-R was corroborated by an abnormal rate of mitophagy events on them [123].

Researchers postulate that mitochondrial dysfunction and CoQ_10_ deficiency could be partly responsible for the pathophysiology of early atherosclerosis by promoting inflammation and enabling increased production of free radicals in the endothelium of blood vessels [125]. The damage infringed by redox imbalance can be extrapolated to other diseases that also involve hyperlipidemia and lipidogenesis, such as diabetes, obesity, and metabolic syndrome [126]. Aiming to ameliorate the conditions of FH patients, treatments targeted at both reducing intracellular cholesterol and raising CoQ_10_ levels should be considered. It has been demonstrated that CoQ_10_ administration can restore increased cholesterol levels and mitochondrial dysfunction in human fibroblasts. Hence, a balanced co-administration of the conventional statin treatment used for hypercholesterolemia patients and CoQ_10_ seems to be the most convenient and accurate approach towards FH therapy [123].

## 3. Recent Formulations

### 3.1. Liposomes

Owing to its remarkable properties as an anti-fatigue, immunostimulating, and antioxidant molecule [127], CoQ_10_ is nowadays widely used as a functional food, drug, and health supplement. Clinically, it is also prescribed for cardiovascular disease, diabetes, viral hepatitis, cancer and many other patients [128]. However, as previously discussed, traditional CoQ_10_ formulations are not satisfactory, since they fail to increase CoQ_10_’s low oral bioavailability. CoQ_10_’s uptake is compromised due to its poor water solubility, instability to light, and thermolability [129]. For this reason, the development of alternative formulations has been a recurrent topic of study in the past few years. Numerous alternatives to the classic CoQ_10_ formulations have been proposed, like solid dispersion systems [130], nanoparticles [131], cyclodextrin inclusion compounds and microcapsules [132]. However, one of the most promising approaches has been the preparation of nano-liposomes with long circulating elements that improve the stability, prolong circulation times, and increase the bioavailability of CoQ_10_ [133]. The main drawback of this liposomal formulation is its high instability. As is widely known, lipid-based vectors are prone to aggregating and forming clustered complexes with larger dimensions [134]. Moreover, the encapsulation efficiency was hindered by the constant leakage of cargo through the lipid layers. In order to implement stability, the lyophilization of liposomes through a freeze-drying procedure was suggested. The lyophilized particles showed stable quality characteristics during long-term storage [89].

### 3.2. Self-Nanoemulsifying Delivery System

CoQ_10_’s low gastrointestinal absorption and low oral bioavailability are a consequence of its low intestinal permeability and high molecular weight. Self-nanoemulsifying CoQ_10_ delivery systems have been developed to face the challenge of this drug’s oral administration by increasing its dissolution in the gastrointestinal tract (GIT) [135]. Self-nanoemulsifying drug delivery systems (SNEDDS) can be defined as an isotropic and thermodynamically stable mixture of an oil, a surfactant, a co-surfactant, and a drug that forms nanoemulsion droplets of a reduced size (<100 nm) when subject to dilution in an aqueous medium under gentle agitation, similar to the gastric movements of the GIT [136]. The fundamental standard of these SNEDDS is that when the emulsion is formed in the GIT of the patient, the drug is presented in a solubilized form inside nano-sized droplets that provide a larger surface area for enhancing the drug’s release and absorption [137]. This novel approach to increase CoQ_10_’s dissolution and absorption has proven successful in recent studies, in which a daily intake of 100 mg of CoQ_10_ SNEDDS is proposed as a sufficient dietary supplement to compensate for low CoQ_10_ levels in patients suffering from various diseases [106].

### 3.3. Novel Lipid-Free Nanoformulation

As previously mentioned, several formulations have been developed to increase CoQ_10_’s bioavailability: an oil solution and a suspension system [138], a lipid and surfactant-based emulsion [139], and a solid dispersion system [140]. However, the bioavailability of CoQ_10_ in these remains low. Thereafter, the next step forward was to develop lipid-free self-emulsifying drug delivery systems (SEDDS) [106], or nanoemulsions [141]. Traditional CoQ_10_ formulations make use of lipid-based delivery systems, because these present several benefits: they increase drug solubility in intestines, recruit lymphatic drug transport or modify enterocyte-based drug transport and disposition [142]. Nevertheless, many other factors like dispersion rate, degree of emulsification, particle size or drug precipitation upon dispersion have a negative impact on the efficacy of lipid-based delivery methods. For this reason, novel studies have focused on the development of lipid-free nano-CoQ_10_ systems. One of the most promising approaches in the field achieved the development of CoQ_10_ nanoformulations with various surfactants but no other lipids. These nano-CoQ_10_ particles were modified with surfactants using hot high-pressure homogenization (HPH) [131]. Such surfactants were intended to alter cell membrane integrity and tight junctions [143] as well as inhibit efflux transporters like P-gp [144] so that permeability would be enhanced. Subsequent studies in rats demonstrated that the orally administered lipid-free nano-CoQ_10_ significantly improved CoQ_10_ bioavailability in comparison to common CoQ_10_ powder suspensions, mainly due to the action of surfactants. Thereby, lipid-free nano-CoQ_10_ complemented with surfactants like PEG40 hydrogenated castor oil (PHCO) or TPGS are a promising alternative for CoQ_10_’s clinical application.

### 3.4. CoQ_10_-Loaded Oleogels

As previously stated, CoQ_10_ is practically insoluble in aqueous solutions, therefore, its oral or intestinal absorption is slow and extremely inefficient. However, its bioavailability can be substantially modified by using an adequate formulation for its administration. Since CoQ_10_ is fat-soluble [145], its absorption is enhanced when taken with a meal having a high oil/fat content. In line with this, oil-based formulations, such as emulsions where CoQ_10_ is dissolved in an oil-dispersed phase, have proven to be successful for CoQ_10_ delivery. The research in the field indicates that solubilized CoQ_10_ formulations present a much higher bioavailability than non-solubilized powder-based CoQ_10_ products [11] meaning that a higher CoQ_10_ plasma concentration could be achieved using lower doses of solubilized CoQ_10_ formulations than those used with non-solubilized ones. Another factor supporting the use of solubilized formulations rather than traditional ones is the fact that mitochondrial and neurodegenerative disorders’ patients commonly struggle to swallow [146,147]. Therefore, it is hard for them to deal with traditionally big CoQ_10_ tablets or powder-filled capsules. In light of this evidence, the research is now focused on developing formulations with CoQ_10_ solubilized either in liquid or jelly matrixes. Among the studies carried out in this direction, it is worth to point out the development of ethyl cellulose (EC)-oleogels for high-dose CoQ_10_ oral administration (1 g of CoQ_10_ per 5 g oleogel-disk) [148]. Medium-chain triglyceride (MCT) oil was used to dissolve CoQ_10_, since it is known to be the only fat that people with the inability to absorb or digest conventional fats tolerate [149]. Moreover, two surfactants were evaluated to modulate the mechanical properties of the gels. SMS proved to be more convenient than lecithin, since it allowed a higher stability to oxidize the MCT oil and a better enhancement of CoQ_10_ stability while lowering the syneresis in the final oleogels. Moreover, SMS-containing oleogels showed higher thermal stability than lecithin-containing ones.

The novelty of the aforementioned study lies in the fact that the SMS-containing oleogels allowed loading exceptionally high doses of soluble CoQ_10_ in soft gel structures that reduce the swallowing discomfort for patients. Additionally, the number of dosage units per day could be reduced since each of these oleogels provides a high dose of CoQ_10_. According to the authors, the CoQ_10_ dissolved in MCT was stable for 12 months when immobilized into the oleogels. Thereafter, neither storage nor distribution is a problem for the future translation of this formulation to the clinical practice.

### 3.5. Novel Water-Soluble CoQ_10_

The use of CoQ_10_ as a functional food is full of promise, since its properties have the ability to beneficially influence body functions, promoting well-being and health as well as reducing the risk of diseases [150]. However, high lipophilicity of this molecule restrains its use as a food-enriching product, especially in aqueous-based preparations. To overcome this barrier, CoQ_10_’s water solubility has been increased by encapsulating it in β-cyclodextrin inclusion complexes [151]. This novel patented formulation is available as Q10Vital and has proved to be stable and well-soluble in diverse aqueous media. A bioequivalence study has confirmed the improved bioavailability and efficacy of this novel CoQ_10_ material in comparison to the traditional soft gel capsules containing CoQ_10_ in soybean oil, which has been the most widely used formulation up to this moment in Europe [152]. According to that study’s results, the mean plasma concentration of CoQ_10_ was highest in the individuals who consumed the liquid Q10Vital formulation, followed by those who took the Q10Vital powder. Moreover, the levels of CoQ_10_ were significantly lower in those who had soft gel capsules. The novel formulation presented higher standard deviations in pharmacokinetics assays owing to its sensitivity to individual pH differences, especially in the gastrointestinal tract. The acidic pH of such an environment may affect the interactions between the guest (CoQ_10_) and the host (β-cyclodextrin) molecules. This inconvenience can be resolved by the co-administration of the formulation with an appropriate food matrix that reduces its pH sensitivity, stabilizes its solution in the GIT, and thereby improves its absorption [117]. All in all, CoQ_10_/β-cyclodextrin complexes have proved to significantly increase water solubilization of CoQ_10_ and, hence, its gastrointestinal absorption. For this reason, this novel formulation, either in its liquid or powder form, represents a more efficient CoQ_10_ delivery method for both the food and pharmaceutical industries.

### 3.6. Micellization of CoQ_10_ by Caspofungin

Since lipophilicity seems to be the main barrier for the parenteral delivery of CoQ_10_, there is a rising interest in the development of water-soluble CoQ_10_ formulations. One of the most famous advances in the field was the formulation of micellar CoQ_10_ nanoparticles [153]. The formation of these hydrophilic particles was mediated by an FDA-approved drug, caspofungin (CF). Despite its moderate surfactant activity, CF successfully solubilizes CoQ_10_, yielding micellar nanoparticles as a result. These nanoparticles were on average smaller than 200 nm in diameter, being therefore ideal for intravenous delivery, since such a reduced size allows for long circulating times and sufficient extravasation and tissue uptake [154,155]. The critical micelle concentration (CMC) is the parameter that reflects the stability of micelles following their dilution in blood. A CMC in the low millimolar range is desirable in drug-carrying compounds, since it indicates high stability after intravenous administration [156]. The CMC of these CF/CoQ_10_ particles is close to 50 µM, indicating that they are unlikely to dissociate rapidly upon injection. According to the authors of the study, the CF/CoQ_10_ formulation can be safely administered to mice via an injection, CoQ_10_ successfully reaching the desired tissues. The highest plasma CoQ_10_ concentration detected following intravenous CF/CoQ_10_ administration (8.6 mg/kg of body weight) was >160 times higher than the endogenous CoQ_9_ level (CoQ_10_ being undetectable). The studied tissues (liver, kidney, heart, skeletal muscle, spleen, lung, and brain) also presented significantly higher CoQ_10_ levels after 10 daily intravenous doses of CF/CoQ_10_ (8.612 mg/kg of body weight). Nonetheless, further research on the uptake differences between tissues should be conducted.

Since both CF and CoQ_10_ are already extensively used in clinical practice and have favorable safety profiles, it is hypothesized that there should not be obstacles for CF/CoQ_10_ micelles’ clinical development and approval for treatment of CoQ_10_ deficiencies and related diseases. However, patients’ discomfort is the main drawback of this therapeutic strategy. As claimed by the authors of the study, CoQ_10_ uptake in organs was cumulative, the reason why several daily injections were required to reach the desired intra-organ CoQ_10_ concentration. Facing the prospect of several injections a day might be an ordeal for many patients [157], the reason why alternative administration procedures should be proposed for this therapy to be unconditionally appealing.

## 4. Conclusions

Coenzyme Q_10_ has always been considered by clinicians and researchers a good ally against a wide range of diseases. Surprising though it may seem, the number of CoQ_10_ applications keeps increasing in our days. Suffice to say that thousands of research teams keep diving into the most remote properties and functions of this molecule, obtaining still surprising results. In the present review, some of the most relevant CoQ_10_ applications have been discussed, as well as the novel formulations for its clinical administration. In addition to its traditional applications (among other diseases, in mitochondrial diseases, Down syndrome, cancer, atherosclerosis, ischemia) CoQ_10_ has proved to be beneficial for diabetic cardiovascular disease and diabetic nephropathy patients, for multiple system atrophy or heart failure treatment, or for epilepsy palliation. Moreover, it has been demonstrated that it can be used as a marker of histological rejection in transplantations or of inflammation. Albeit its most remarkable newborn applications are its protective properties against UV radiation, its highly beneficial effect on familial hypercholesterolemia patients and its neuroprotective potential when administered together with TPGS.

Being aware of CoQ_10_’s clinical interest, numerous research groups have developed promising formulations to ensure a more efficient administration of this drug. Among them, the development of a novel water-soluble CoQ_10_ form should be highlighted. These new delivery systems are intended to overcome the great barrier that prevents CoQ_10_ from becoming a widely used pharmaceutical drug and food complement: its low oral bioavailability.

The unceasing increase in the number of CoQ_10_ applications is flabbergasting, to say the least. Were the bottleneck of its delivery to be overcome by the newly developed formulations, CoQ_10_ would become one of the most widely used molecules for therapeutic purposes.

## Figures and Tables

**Table 1:** Novel medical applications of CoQ_10_.

Disease	CoQ_10_ Application	References
Diabetic cardiovascular diseases and diabetic nephropathy	Significant reduction in adverse cardiovascular events	[11,15,16,17,18,19,20,21,22]
Multiple system atrophy	Accurate biomarker for MSA when found in low levels in cerebrospinal fluid	[20,23,24,25,26,27,28,29,30]
Immunological disorders	Significant improvement of the T cell function Reduction of circulating inflammatory markers	[14,31,32,33,34,35,36,37,38,39,40,41,42,43,44,45,46,47,48,49,50]
Neuropathies	Neuroprotection via Ca_2_^+^ buffering and antioxidant activity	[35,51,52,53,54,55,56,57,58,59,60,61,62,63,64,65,66,67,68,69,70,71,72,73,74,75,76,77,78,79]
UVB radiation damage	Treatment of damaged skin	[66,80,81,82,83,84,85]
Heart failure	Supplementation required for improving patient condition	[82,86,87,88,89,90,91,92,93]
Barth syndrome and membrane instability-related diseases	CoQ_10_-based treatment for Barth syndrome patients Development of cholesterol-free liposome-based drug delivery systems	[94,95,96,97,98,99,100,101,102,103,104,105,106]
Insulin resistance	Increase in insulin sensitivity by scavenging mitochondrial oxidants	[107,108,109,110,111,112,113,114,115]
Fibromyalgia	Pain alleviation	[116,117,118,119,120,121]
Familial hypercholesterolemia and atherosclerosis	Supplementation required for improving patient condition	[122,123,124,125,126]

## Abbreviations

CoQ_10_	Coenzyme Q_10_
ADI	Acceptable daily intake
NOAEL	No-observed-adverse-effect level
UVB	Ultraviolet B
CVD	Diabetic cardiovascular disease
ROS	Reactive oxygen species
RAGE	Receptor for advanced glycation end products
AGEs	Advanced glycation end products
NADPH	Nicotinamide adenine dinucleotide phosphate
PKC	Protein kinase C
DN	Diabetic nephropathy
UTMD	Ultrasound-targeted microbubble destruction
MSA	Multiple system atrophy
CSF	Cerebrospinal fluid
COQ2	Coenzyme Q2
COQ9	Coenzyme Q9
COQ8A	Coenzyme Q8A
ETFDH	Electron transfer flavoprotein dehydrogenase
PDSS2	Decaprenyl diphosphate synthase subunit 2
AIDS	Acquired immune deficiency syndrome
CRP	C-reactive protein
IL-6	Interleukin 6
TNF-α	Tumor necrosis factor alpha
P-gp	P-glycoprotein
TPGS	α-tocopherol polyethylene glycol 1000 succinate
RGC	Retinal ganglion cells
IOP	Intraocular pressure
PD	Parkinson disease
NF-β	Nuclear factor beta
POPC	Palmitoyl-2-oleoyl-sn-glycero-3-phosphocholine
IMM	Inner mitochondrial membrane
IIF	Flavin site in complex II
FM	Fibromyalgia
IL-1β	Interleukin 1 beta
UV	Ultraviolet
IL-18	Interleukin 18
FH	Familial hypercholesterolemia
GIT	Gastrointestinal tract
SNEDDS	Self-nanoemulsifying drug delivery systems
HPH	High-pressure homogenization
PHCO	PEG40 hydrogenated castor oil
TPGS	Tocopherol polyethylene glycol succinate
EC	Ethyl cellulose
MCT	Medium-chain triglyceride
SMS	SMS detergent
CMC	Critical micelle concentration
HMGCR	3-hydroxy-3-methyl-glutaryl-coenzyme A reductase
LDL	Low-density lipoprotein
LDL-R	Low-density lipoprotein receptor
ACE	Angiotensin-converting enzyme
CHF	Chronic heart failure

## References

[1] Festenstein G.N., Heaton F.W., Lowe J.S., Morton R.A. (1955). A constituent of the unsaponifiable portion of animal tissue lipids (lambda max. 272 m mu). Biochem. J..

[2] Crane F.L., Hatefi Y., Lester R.L., Widmer C. (1957). Isolation of a quinone from beef heart mitochondria. Biochim. Biophys. Acta.

[3] Overvad K., Diamant B., Holm L., Hùlmer G., Mortensen S.A., Stender S. (1999). Review Coenzyme Q 10 in health and disease. Eur. J. Clin. Nutr..

[4] Tacchino F., Succurro A., Ebenhöh O., Gerace D. (2019). Optimal efficiency of the Q-cycle mechanism around physiological temperatures from an open quantum systems approach. Sci. Rep..

[5] Nelson D.L., Cox M.M., Lehninger A.L. (2017). Lehninger Principles of Biochemistry.

[6] Ernster L., Dallner G. (1995). Biochemical, physiological and medical aspects of ubiquinone function. Biochim. Biophys. Acta (BBA) Mol. Basis Dis..

[7] Parikh S., Saneto R., Falk M.J., Anselm I., Cohen B.H., Haas R., Medicine Society T.M. (2009). A modern approach to the treatment of mitochondrial disease. Curr. Treat. Options Neurol..

[8] Groneberg D.A., Kindermann B., Althammer M., Klapper M., Vormann J., Littarru G.P., Döring F. (2005). Coenzyme Q10 affects expression of genes involved in cell signalling, metabolism and transport in human CaCo-2 cells. Int. J. Biochem. Cell Biol..

[9] Crane F.L. (1999). New functions for CoenzymeQ10. Protoplasma.

[10] Alleva R., Tomasetti M., Andera L., Gellert N., Borghi B., Weber C., Murphy M.P., Neuzil J. (2001). Coenzyme Q blocks biochemical but not receptor-mediated apoptosis by increasing mitochondrial antioxidant protection. FEBS Lett..

[11] Bhagavan H.N., Chopra R.K. (2007). Plasma coenzyme Q10 response to oral ingestion of coenzyme Q10 formulations. Mitochondrion.

[12] Hidaka T., Fujii K., Funahashi I., Fukutomi N., Hosoe K. (2008). Safety assessment of coenzyme Q 10 (CoQ 10). BioFactors.

[13] Rodríguez-Hernández Á., Cordero M.D., Salviati L., Artuch R., Pineda M., Briones P., Izquierdo L.G., Cotán D., Navas P., Sánchez-Alcázar J.A. (2009). Coenzyme Q deficiency triggers mitochondria degradation by mitophagy. Autophagy.

[14] Farough S., Karaa A., Walker M.A., Slate N., Dasu T., Verbsky J., Fusunyan R., Canapari C., Kinane T.B., Van Cleave J. (2014). Coenzyme Q10 and immunity: A case report and new implications for treatment of recurrent infections in metabolic diseases. Clin. Immunol..

[15] Fox C.S., Coady S., Sorlie P.D., D’Agostino R.B., Pencina M.J., Vasan R.S., Meigs J.B., Levy D., Savage P.J. (2007). Increasing cardiovascular disease burden due to diabetes mellitus: The Framingham Heart Study. Circulation.

[16] Faria A., Persaud S.J. (2017). Cardiac oxidative stress in diabetes: Mechanisms and therapeutic potential. Pharmacol. Ther..

[17] Mortensen S.A., Rosenfeldt F., Kumar A., Dolliner P., Filipiak K.J., Pella D., Alehagen U., Steurer G., Littarru G.P. (2014). The effect of coenzyme Q10on morbidity and mortality in chronic heart failure: Results from Q-SYMBIO: A randomized double-blind trial. JACC Heart Fail..

[18] Yue T., Xu H.L., Chen P.P., Zheng L., Huang Q., Sheng W.S., Zhuang Y.D., Jiao L.Z., Chi T.T., ZhuGe D.L. (2017). Combination of coenzyme Q10-loaded liposomes with ultrasound targeted microbubbles destruction (UTMD) for early theranostics of diabetic nephropathy. Int. J. Pharm..

[19] Mima A. (2013). Inflammation and oxidative stress in diabetic nephropathy: New insights on its inhibition as new therapeutic targets. J. Diabetes Res..

[20] Brownlee M. (2001). Biochemicstry and molecular cell biology of diabetic complications. Nature.

[21] ElBayoumi T.A., Torchilin V.P., Weissig V. (2010). Current Trends in Liposome Research BT-Liposomes: Methods and Protocols, Volume 1: Pharmaceutical Nanocarriers.

[22] Zhang Y., Ye C., Xu Y., Dong X., Li J., Liu R., Gao Y. (2014). Ultrasound-mediated microbubble destruction increases renal interstitial capillary permeability in early diabetic nephropathy rats. Ultrasound Med. Biol..

[23] Fanciulli A., Wenning G.K. (2015). Multiple-System Atrophy. N. Engl. J. Med..

[24] Hughes A.J., Daniel S.E., Ben-Shlomo Y., Lees A.J. (2002). The accuracy of diagnosis of parkinsonian syndromes in a specialist movement disorder service. Brain.

[25] Koga S., Aoki N., Uitti R.J., Van Gerpen J.A., Cheshire W.P., Josephs K.A., Wszolek Z.K., Langston J.W., Dickson D.W. (2015). When DLB, PD, and PSP masquerade as MSA. Neurology.

[26] Kasai T., Tokuda T., Ohmichi T., Ishii R., Tatebe H., Nakagawa M., Mizuno T. (2016). Serum Levels of Coenzyme Q10 in Patients with Multiple System Atrophy. PLoS ONE.

[27] Mitsui J., Matsukawa T., Yasuda T., Ishiura H., Tsuji S. (2016). Plasma coenzyme q10 levels in patients with multiple system atrophy. JAMA Neurol..

[28] Compta Y., Giraldo D.M., Muñoz E., Antonelli F., Fernández M., Bravo P., Soto M., Cámara A., Torres F., Martí M.J. (2018). Cerebrospinal fluid levels of coenzyme Q10 are reduced in multiple system atrophy. Parkinsonism Relat. Disord..

[29] American N. (2013). Mutations in COQ2 in Familial and Sporadic Multiple-System Atrophy. N. Engl. J. Med..

[30] Mitsui J., Koguchi K., Momose T., Takahashi M., Matsukawa T., Yasuda T., Tokushige S.I., Ishiura H., Goto J., Nakazaki S. (2017). Three-Year Follow-Up of High-Dose Ubiquinol Supplementation in a Case of Familial Multiple System Atrophy with Compound Heterozygous COQ2 Mutations. Cerebellum.

[31] Rötig A., Appelkvist E.-L., Geromel V., Chretien D., Kadhom N., Edery P., Lebideau M., Dallner G., Munnich A., Ernster L. (2018). Quinone-responsive multiple respiratory-chain dysfunction due to widespread coenzyme Q10 deficiency. Lancet.

[32] Bird L. (2019). Getting enough energy for immunity. Nat. Rev. Immunol..

[33] Arias A., García-Villoria J., Rojo A., Buján N., Briones P., Ribes A. (2012). Analysis of coenzyme Q10 in lymphocytes by HPLC–MS/MS. J. Chromatogr. B.

[34] Folkers K., Hanioka T., Xia L.-J., McRee J.T., Langsjoen P. (1991). Coenzyme Q10 increases T4/T8 ratios of lymphocytes in ordinary subjects and relevance to patients having the aids related complex. Biochem. Biophys. Res. Commun..

[35] Folkers K., Morita M., McRee J. (1993). The Activities of Coenzyme Q10 and Vitamin B6 for Immune Responses. Biochem. Biophys. Res. Commun..

[36] Fan L., Feng Y., Chen G.C., Qin L.Q., Fu C.l., Chen L.H. (2017). Effects of coenzyme Q10 supplementation on inflammatory markers: A systematic review and meta-analysis of randomized controlled trials. Pharm. Res..

[37] Herder C., Illig T., Rathmann W., Martin S., Haastert B., Mller-Scholze S., Holle R., Thorand B., Koenig W., Wichmann H.E. (2005). Inflammation and type 2 diabetes: Results from KORA Augsburg. Gesundheitswesen.

[38] Libby P. (2006). Inflammation and cardiovascular disease mechanisms. Am. J. Clin. Nutr..

[39] Tudorache E., Oancea C., Avram C., Fira-Mladinescu O., Petrescu L., Timar B. (2015). Balance impairment and systemic inflammation in chronic obstructive pulmonary disease. Int. J. Chronic Obstr. Pulm. Dis..

[40] Coussens L.M., Werb Z. (2002). Inflammation and cancer. Nature.

[41] Rosner M.H., Ronco C., Okusa M.D. (2018). The Role of Inflammation in the Cardio-Renal Syndrome: A Focus on Cytokines and Inflammatory Mediators. Semin. Nephrol..

[42] Tarry-Adkins J.L., Fernandez-Twinn D.S., Hargreaves I.P., Neergheen V., Aiken C.E., Martin-Gronert M.S., McConnell J.M., Ozanne S.E. (2016). Coenzyme Q10 prevents hepatic fibrosis, inflammation, and oxidative stress in a male rat model of poor maternal nutrition and accelerated postnatal growth1. Am. J. Clin. Nutr..

[43] Hernández-Camacho J.D., Bernier M., López-Lluch G., Navas P. (2018). Coenzyme Q(10) Supplementation in Aging and Disease. Front. Physiol..

[44] Haidari M., Javadi E., Sadeghi B., Hajilooi M., Ghanbili J. (2001). Evaluation of C-reactive protein, a sensitive marker of inflammation, as a risk factor for stable coronary artery disease. Clin. Biochem..

[45] Nakanishi S., Yamane K., Kamei N., Okubo M., Kohno N. (2003). Elevated C-Reactive Protein Is a Risk Factor for the Development of Type 2 Diabetes in Japanese Americans. Diabetes Care.

[46] Oh D.J., Kim H.R., Lee M.K., Woo Y.S. (2013). Proflie of Human β-Defensins 1,2 and Proinflammatory Cytokines (TNF-α, IL-6) in Patients with Chronic Kidney Disease. Kidney Blood Press. Res..

[47] Saxena M., Srivastava N., Banerjee M. (2013). Association of IL-6, TNF-α and IL-10 gene polymorphisms with type 2 diabetes mellitus. Mol. Biol. Rep..

[48] Liu Y. (2006). IL-6 Haplotypes, Inflammation, and Risk for Cardiovascular Disease in a Multiethnic Dialysis Cohort. J. Am. Soc. Nephrol..

[49] Ebadi M., Sharma S.K., Wanpen S., Amornpan A. (2004). Coenzyme Q10 inhibits mitochondrial complex-1 down-regulation and nuclear factor-kappa B activation. J. Cell. Mol. Med..

[50] Schmelzer C., Lindner I., Rimbach G., Niklowitz P., Menke T., Döring F. (2008). Functions of coenzyme Q10 in inflammation and gene expression. BioFactors.

[51] Amor S., Puentes F., Baker D., Van Der Valk P. (2010). Inflammation in neurodegenerative diseases. Immunology.

[52] Yacoubian T.A., Standaert D.G. (2009). Targets for neuroprotection in Parkinson’s disease. Biochim. Biophys. Acta-Mol. Basis Dis..

[53] Alam Z.I., Jenner A., Daniel S.E., Lees A.J., Cairns N., Marsden C.D., Jenner P., Halliwell B. (1997). Oxidative DNA damage in the parkinsonian brain: An apparent selective increase in 8-hydroxyguanine levels in substantia nigra. J. Neurochem..

[54] Hastings T.G., Lewis D.A., Zigmond M.J., Snyder R., Sipes I.G., Jollow D.J., Monks T.J., Kocsis J.J., Kalf G.F., Greim H., Witmer C.M. (1996). Reactive Dopamine Metabolites and Neurotoxicity BT-Biological Reactive Intermediates V: Basic Mechanistic Research in Toxicology and Human Risk Assessment.

[55] Schapira A.H.V., Cooper J.M., Dexter D., Jenner P., Clark J.B., Marsden C.D. (2018). Mitochondrial Complex I Deficiency in Parkinson’s Disease. Lancet.

[56] Glickman R.D. (2011). Ultraviolet phototoxicity to the retina. Eye Contact Lens.

[57] Zhang X., Tohari A.M., Marcheggiani F., Zhou X., Reilly J., Tiano L., Shu X. (2017). Therapeutic Potential of Co-enzyme Q10 in Retinal Diseases. Curr. Med. Chem..

[58] Qu J., Kaufman Y., Washington I. (2009). Coenzyme Q10 in the Human Retina. Investig. Opthalmol. Vis. Sci..

[59] Martucci A., Nucci C. (2019). Evidence on neuroprotective properties of coenzyme Q10 in the treatment of glaucoma. Neural Regen Res..

[60] Lulli M., Witort E., Papucci L., Torre E., Schipani C., Bergamini C., Dal Monte M., Capaccioli S. (2012). Coenzyme Q10 Instilled as Eye Drops on the Cornea Reaches the Retina and Protects Retinal Layers from Apoptosis in a Mouse Model of Kainate-Induced Retinal Damage. Investig. Ophthalmol. Visual Sci..

[61] Davis B.M., Tian K., Pahlitzsch M., Brenton J., Ravindran N., Butt G., Malaguarnera G., Normando E.M., Guo L., Cordeiro M.F. (2017). Topical Coenzyme Q10 demonstrates mitochondrial-mediated neuroprotection in a rodent model of ocular hypertension. Mitochondrion.

[62] Itagaki S., Ochiai A., Kobayashi M., Sugawara M., Hirano T., Iseki K. (2008). Interaction of Coenzyme Q10 with the Intestinal Drug Transporter P-Glycoprotein. J. Agric. Food Chem..

[63] Fato R., Bergamini C., Leoni S., Pinna A., Carta F., Cardascia N., Ferrari T.M., Sborgia C., Lenaz G. (2010). Coenzyme Q10 vitreous levels after administration of coenzyme Q10 eyedrops in patients undergoing vitrectomy. Acta Ophthalmol..

[64] Nakajima Y., Inokuchi Y., Nishi M., Shimazawa M., Otsubo K., Hara H. (2008). Coenzyme Q10 protects retinal cells against oxidative stress in vitro and in vivo. Brain Res..

[65] Beal M.F. (2004). Therapeutic Effects of Coenzyme Q10 in Neurodegenerative Diseases. Methods in Enzymology.

[66] Turunen M., Olsson J., Dallner G. (2004). Metabolism and function of coenzyme Q. Biochim. Biophys. Acta (BBA) Biomembr..

[67] Bogeski I., Gulaboski R., Kappl R., Mirceski V., Stefova M., Petreska J., Hoth M. (2011). Calcium Binding and Transport by Coenzyme Q. J. Am. Chem. Soc..

[68] Pinton P., Giorgi C., Siviero R., Zecchini E., Rizzuto R. (2008). Calcium and apoptosis: ER-mitochondria Ca^2+^ transfer in the control of apoptosis. Oncogene.

[69] Weinreb R.N., Khaw P.T. (2004). Primary open-angle glaucoma. Lancet.

[70] Chrysostomou V., Rezania F., Trounce I.A., Crowston J.G. (2013). Oxidative stress and mitochondrial dysfunction in glaucoma. Curr. Opin. Pharm..

[71] Osborne N.N., del Olmo-Aguado S. (2013). Maintenance of retinal ganglion cell mitochondrial functions as a neuroprotective strategy in glaucoma. Curr. Opin. Pharmacol..

[72] Nucci C., Tartaglione R., Cerulli A., Mancino R., Spanò A., Cavaliere F., Rombolà L., Bagetta G., Corasaniti M.T., Morrone L.A. (2007). Retinal Damage Caused by High Intraocular Pressure–Induced Transient Ischemia is Prevented by Coenzyme Q10 in Rat. International Review of Neurobiology.

[73] Russo R., Cavaliere F., Rombolà L., Gliozzi M., Cerulli A., Nucci C., Fazzi E., Bagetta G., Corasaniti M.T., Morrone L.A., Nucci C., Cerulli L., Osborne N.N., Bagetta G. (2008). Rational basis for the development of coenzyme Q10 as a neurotherapeutic agent for retinal protection. Progress in Brain Research.

[74] Lee D., Shim M.S., Kim K.-Y., Noh Y.H., Kim H., Kim S.Y., Weinreb R.N., Ju W.-K. (2014). Coenzyme Q10 inhibits glutamate excitotoxicity and oxidative stress-mediated mitochondrial alteration in a mouse model of glaucoma. Investig. Ophthalmol. Visual Sci..

[75] Fernández-Vega B., Nicieza J., Álvarez-Barrios A., Álvarez L., García M., Fernández-Vega C., Vega J.A., González-Iglesias H. (2020). The Use of Vitamins and Coenzyme Q10 for the Treatment of Vascular Occlusion Diseases Affecting the Retina. Nutrients.

[76] Bhardwaj M., Kumar A. (2016). Neuroprotective mechanism of Coenzyme Q10 (CoQ10) against PTZ induced kindling and associated cognitive dysfunction: Possible role of microglia inhibition. Pharmacol. Rep..

[77] Lucas S.-M., Rothwell N.J., Gibson R.M. (2009). The role of inflammation in CNS injury and disease. Br. J. Pharm..

[78] Papucci L., Schiavone N., Witort E., Donnini M., Lapucci A., Tempestini A., Formigli L., Zecchi-Orlandini S., Orlandini G., Carella G. (2003). Coenzyme Q10 prevents apoptosis by inhibiting mitochondrial depolarization independently of its free radical scavenging property. J. Biol. Chem..

[79] Mancuso M., Orsucci D., Calsolaro V., Choub A., Siciliano G. (2009). Coenzyme Q10 and Neurological Diseases. Pharmaceuticals.

[80] Al Shaal L., Shegokar R., Müller R.H. (2011). Production and characterization of antioxidant apigenin nanocrystals as a novel UV skin protective formulation. Int. J. Pharm..

[81] Valko M., Leibfritz D., Moncol J., Cronin M.T.D., Mazur M., Telser J. (2007). Free radicals and antioxidants in normal physiological functions and human disease. Int. J. Biochem. Cell Biol..

[82] Mukherjee P.K., Maity N., Nema N.K., Sarkar B.K. (2011). Bioactive compounds from natural resources against skin aging. Phytomedicine.

[83] Pegoraro N.S., Barbieri A.V., Camponogara C., Mattiazzi J., Brum E.S., Marchiori M.C.L., Oliveira S.M., Cruz L. (2017). Nanoencapsulation of coenzyme Q10 and vitamin E acetate protects against UVB radiation-induced skin injury in mice. Colloids Surf. B Biointerfaces.

[84] Gupta S., Gupta S., Jindal N., Jindal A., Bansal R. (2013). Nanocarriers and nanoparticles for skin care and dermatological treatments. Indian Dermatol. Online J..

[85] Osmałek T., Froelich A., Tasarek S. (2014). Application of gellan gum in pharmacy and medicine. Int. J. Pharm..

[86] Kumar A., Kaur H., Devi P., Mohan V. (2009). Role of coenzyme Q10 (CoQ10) in cardiac disease, hypertension and Meniere-like syndrome. Pharm. Ther..

[87] Linnane A.W., Kopsidas G., Zhang C., Yarovaya N., Kovalenko S., Papakostopoulos P., Eastwood H., Graves S., Richardson M. (2002). Cellular Redox Activity of Coenzyme Q 10: Effect of CoQ 10 Supplementation on Human Skeletal Muscle. Free Radic. Res..

[88] Folkers K., Vadhanavikit S., Mortensen S.A. (1985). Biochemical rationale and myocardial tissue data on the effective therapy of cardiomyopathy with coenzyme Q10. Proc. Natl. Acad. Sci. USA.

[89] Belardinelli R., Muçaj A., Lacalaprice F., Solenghi M., Seddaiu G., Principi F., Tiano L., Littarru G.P. (2006). Coenzyme Q10 and exercise training in chronic heart failure. Eur. Heart J..

[90] Di Lorenzo A., Iannuzzo G., Parlato A., Cuomo G., Testa C., Coppola M., D’Ambrosio G., Oliviero D.A., Sarullo S., Vitale G. (2020). Clinical Evidence for Q10 Coenzyme Supplementation in Heart Failure: From Energetics to Functional Improvement. J. Clin. Med..

[91] Jankowski J., Korzeniowska K., Cieślewicz A., Jabłecka A. (2016). Coenzyme Q10–A new player in the treatment of heart failure?. Pharmacol. Rep..

[92] Molyneux S.L., Florkowski C.M., George P.M., Pilbrow A.P., Frampton C.M., Lever M., Richards A.M. (2008). Coenzyme Q10: An Independent Predictor of Mortality in Chronic Heart Failure. J. Am. Coll. Cardiol..

[93] Gvozdjáková A., Kucharská J., Mizera S., Braunová Z., Schreinerová Z., Schrameková E., Pecháň I., Fabián J. (1999). Coenzyme Q10 depletion and mitochondrial energy disturbances in rejection development in patients after heart transplantation. BioFactors.

[94] Barth P.G., Scholte H.R., Berden J.A., Van Der Klei-Van Moorsel J.M., Luyt-Houwen I.E.M., Van’T Veer-Korthof E.T., Van Der Harten J.J., Sobotka-Plojhar M.A. (1983). An X-linked mitochondrial disease affecting cardiac muscle, skeletal muscle and neutrophil leucocytes. J. Neurol. Sci..

[95] Schlame M., Ren M. (2006). Barth syndrome, a human disorder of cardiolipin metabolism. FEBS Lett..

[96] Schlame M., Rua D., Greenberg M.L. (2000). The biosynthesis and functional role of cardiolipin. Prog. Lipid Res..

[97] Chicco A.J., Sparagna G.C. (2007). Role of cardiolipin alterations in mitochondrial dysfunction and disease. Am. J. Physiol. Cell Physiol..

[98] Paradies G., Ruggiero F.M. (1988). Effect of hyperthyroidism on the transport of pyruvate in rat-heart mitochondria. Biochim. Biophys. Acta (BBA) Bioenerg..

[99] Paradies G., Ruggiero F.M., Dinoi P. (1991). The influence of hypothyroidism on the transport of phosphate and on the lipid composition in rat-liver mitochondria. Biochim. Biophys. Acta (BBA) Biomembr..

[100] Lee H.-J., Mayette J., Rapoport S.I., Bazinet R.P. (2006). Selective remodeling of cardiolipin fatty acids in the aged rat heart. Lipids Health Dis..

[101] Sparagna G.C., Johnson C.A., McCune S.A., Moore R.L., Murphy R.C. (2005). Quantitation of cardiolipin molecular species in spontaneously hypertensive heart failure rats using electrospray ionization mass spectrometry. J. Lipid Res..

[102] Paradies G., Petrosillo G., Gadaleta M.N., Ruggiero F.M. (1999). The effect of aging and acetyl-l-carnitine on the pyruvate transport and oxidation in rat heart mitochondria. FEBS Lett..

[103] Petrosillo G., Francesca M.R., Di Venosa N., Paradies A.G. (2003). Decreased complex III activity in mitochondria isolated from rat heart subjected to ischemia and reperfusion: Role of reactive oxygen species and cardiolipin. FASEB J..

[104] Yan N., Liu Y., Liu L., Du Y., Liu X., Zhang H., Zhang Z. (2019). Bioactivities and Medicinal Value of Solanesol and Its Accumulation, Extraction Technology, and Determination Methods. Biomolecules.

[105] Kaurola P., Sharma V., Vonk A., Vattulainen I., Róg T. (2016). Distribution and dynamics of quinones in the lipid bilayer mimicking the inner membrane of mitochondria. Biochim. Biophys. Acta (BBA) Biomembr..

[106] Agmo Hernández V., Eriksson E.K., Edwards K. (2015). Ubiquinone-10 alters mechanical properties and increases stability of phospholipid membranes. Biochim. Biophys. Acta Biomembr..

[107] Hoehn K.L., Salmon A.B., Hohnen-Behrens C., Turner N., Hoy A.J., Maghzal G.J., Stocker R., Van Remmen H., Kraegen E.W., Cooney G.J. (2009). Insulin resistance is a cellular antioxidant defense mechanism. Proc. Natl. Acad. Sci. USA.

[108] Anderson E.J., Lustig M.E., Boyle K.E., Woodlief T.L., Kane D.A., Lin C.-T., Price Iii J.W., Kang L., Rabinovitch P.S., Szeto H.H. (2009). Mitochondrial H2O2 emission and cellular redox state link excess fat intake to insulin resistance in both rodents and humans. J. Clin. Investig..

[109] Fazakerley D.J., Chaudhuri R., Yang P., Maghzal G.J., Thomas K.C., Krycer J.R., Humphrey S.J., Parker B.L., Fisher-Wellman K.H., Meoli C.C. (2018). Mitochondrial CoQ deficiency is a common driver of mitochondrial oxidants and insulin resistance. eLife.

[110] Quinlan C.L., Orr A.L., Perevoshchikova I.V., Treberg J.R., Ackrell B.A., Brand M.D. (2012). Mitochondrial complex II can generate reactive oxygen species at high rates in both the forward and reverse reactions. J. Biol. Chem..

[111] Zhang Y., Åberg F., Appelkvist E.-L., Dallner G., Ernster L. (1995). Uptake of Dietary Coenzyme Q Supplement is Limited in Rats. J. Nutr..

[112] Brand M.D., Goncalves R.L.S., Orr A.L., Vargas L., Gerencser A.A., Borch Jensen M., Wang Y.T., Melov S., Turk C.N., Matzen J.T. (2016). Suppressors of Superoxide-H(2)O(2) Production at Site I(Q) of Mitochondrial Complex I Protect against Stem Cell Hyperplasia and Ischemia-Reperfusion Injury. Cell Metab..

[113] Orr A.L., Vargas L., Turk C.N., Baaten J.E., Matzen J.T., Dardov V.J., Attle S.J., Li J., Quackenbush D.C., Goncalves R.L.S. (2015). Suppressors of superoxide production from mitochondrial complex III. Nat. Chem. Biol..

[114] Cederberg H., Stančáková A., Yaluri N., Modi S., Kuusisto J., Laakso M. (2015). Increased risk of diabetes with statin treatment is associated with impaired insulin sensitivity and insulin secretion: A 6 year follow-up study of the METSIM cohort. Diabetologia.

[115] Preiss D., Seshasai S., Welsh P., Murphy S.A., Ho J.E., Waters D.D., Demicco D.A., Barter P., Cannon C.P., Sabatine M.S. (2011). Risk of incident diabetes with intensive-dose compared with moderate-dose statin therapy: A meta-analysis. JAMA.

[116] Cordero M.D., Moreno-Fernández A.M., de Miguel M., Bonal P., Campa F., Jiménez-Jiménez L.M., Ruiz-Losada A., Sánchez-Domínguez B., Sánchez Alcázar J.A., Salviati L. (2009). Coenzyme Q10 distribution in blood is altered in patients with Fibromyalgia. Clin. Biochem..

[117] Cordero M.D., De Miguel M., Moreno Fernández A.M., Carmona López I.M., Garrido Maraver J., Cotán D., Gómez Izquierdo L., Bonal P., Campa F., Bullon P. (2010). Mitochondrial dysfunction and mitophagy activation in blood mononuclear cells of fibromyalgia patients: Implications in the pathogenesis of the disease. Arthritis Res. Ther..

[118] Bagis S., Tamer L., Sahin G., Bilgin R., Guler H., Ercan B., Erdogan C. (2005). Free radicals and antioxidants in primary fibromyalgia: An oxidative stress disorder?. Rheumatol. Int..

[119] Cordero M.D., Alcocer-Gómez E., Culic O., Carrión A.M., de Miguel M., Díaz-Parrado E., Pérez-Villegas E.M., Bullón P., Battino M., Sánchez-Alcazar J.A. (2014). NLRP3 Inflammasome Is Activated in Fibromyalgia: The Effect of Coenzyme Q10. Antioxid. Redox Signal..

[120] Menu P., Vince J.E. (2011). The NLRP3 inflammasome in health and disease: The good, the bad and the ugly. Clin. Exp. Immunol..

[121] Cordero M.D., Díaz-Parrado E., Carrión A.M., Alfonsi S., Sánchez-Alcazar J.A., Bullón P., Battino M., de Miguel M. (2013). Is Inflammation a Mitochondrial Dysfunction-Dependent Event in Fibromyalgia?. Antioxid. Redox Signal..

[122] Avogaro P., Cazzolato G. (1975). Familial hyper-HDL-(α)-cholesterolemia. Atherosclerosis.

[123] Suárez-Rivero J.M., de la Mata M., Pavón A.D., Villanueva-Paz M., Povea-Cabello S., Cotán D., Álvarez-Córdoba M., Villalón-García I., Ybot-González P., Salas J.J. (2018). Intracellular cholesterol accumulation and coenzyme Q10 deficiency in Familial Hypercholesterolemia. Biochim. Biophys. Acta (BBA) Mol. Basis Dis..

[124] Griffin S., Preta G., Sheldon I.M. (2017). Inhibiting mevalonate pathway enzymes increases stromal cell resilience to a cholesterol-dependent cytolysin. Sci. Rep..

[125] Marzetti E., Csiszar A., Dutta D., Balagopal G., Calvani R., Leeuwenburgh C. (2013). Role of mitochondrial dysfunction and altered autophagy in cardiovascular aging and disease: From mechanisms to therapeutics. Am. J. Physiol. Heart Circ. Physiol..

[126] Vercesi A.E., Castilho R.F., Kowaltowski A.J., Oliveira H.C. (2007). Mitochondrial energy metabolism and redox state in dyslipidemias. IUBMB Life.

[127] Mohammadi-Bardbori A., Najibi A., Amirzadegan N., Gharibi R., Dashti A., Omidi M., Saeedi A., Ghafarian-Bahreman A., Niknahad H. (2015). Coenzyme Q10 remarkably improves the bio-energetic function of rat liver mitochondria treated with statins. Eur. J. Pharmacol..

[128] Villalba J.M., Parrado C., Santos-Gonzalez M., Alcain F.J. (2010). Therapeutic use of coenzyme Q10 and coenzyme Q10-related compounds and formulations. Expert Opin. Investig. Drugs.

[129] Yamada Y., Nakamura K., Abe J., Hyodo M., Haga S., Ozaki M., Harashima H. (2015). Mitochondrial delivery of Coenzyme Q10 via systemic administration using a MITO-Porter prevents ischemia/reperfusion injury in the mouse liver. J. Control. Release.

[130] Onoue S., Uchida A., Kuriyama K., Nakamura T., Seto Y., Kato M., Hatanaka J., Tanaka T., Miyoshi H., Yamada S. (2012). Novel solid self-emulsifying drug delivery system of coenzyme Q10 with improved photochemical and pharmacokinetic behaviors. Eur. J. Pharm. Sci..

[131] Zhou H., Liu G., Zhang J., Sun N., Duan M., Yan Z., Xia Q. (2014). Novel lipid-free nanoformulation for improving oral bioavailability of coenzyme Q10. BioMed Res. Int..

[132] Beg S., Javed S., Kohli K. (2010). Bioavailability enhancement of coenzyme Q10: An extensive review of patents. Recent Patents Drug Deliv. Formul..

[133] Li H., Chen F. (2017). Preparation and quality evaluation of coenzyme Q10 long-circulating liposomes. Saudi J. Biol. Sci..

[134] Yang S., Chen J., Zhao D., Han D., Chen X. (2012). Comparative study on preparative methods of DC-Chol/DOPE liposomes and formulation optimization by determining encapsulation efficiency. Int. J. Pharm..

[135] Pund S., Shete Y., Jagadale S. (2014). Multivariate analysis of physicochemical characteristics of lipid based nanoemulsifying cilostazol—Quality by design. Colloids Surf. B Biointerfaces.

[136] Khattab A., Hassanin L., Zaki N. (2017). Self-Nanoemulsifying Drug Delivery System of Coenzyme (Q10) with Improved Dissolution, Bioavailability, and Protective Efficiency on Liver Fibrosis. AAPS PharmSciTech.

[137] Singh A., Singh V., Juyal D., Rawat G. (2015). Self emulsifying systems: A review. Asian J. Pharm..

[138] Ullmann U., Metzner J., Schulz C., Perkins J., Leuenberger B. (2005). A new coenzyme Q10 tablet-grade formulation (all-Q (R)) is bioequivalent to Q-Gel (R) and both have better bioavailability properties than Q-SorB (R). J. Med. Food.

[139] Thanatuksorn P., Kawai K., Hayakawa M., Hayashi M., Kajiwara K. (2009). Improvement of the oral bioavailability of coenzyme Q10 by emulsification with fats and emulsifiers used in the food industry. LWT-Food Sci. Technol..

[140] Nepal P.R., Han H.-K., Choi H.-K. (2010). Enhancement of solubility and dissolution of Coenzyme Q10 using solid dispersion formulation. Int. J. Pharm..

[141] Belhaj N., Dupuis F., Arab-Tehrany E., Denis F.M., Paris C., Lartaud I., Linder M. (2012). Formulation, characterization and pharmacokinetic studies of coenzyme Q10 PUFA’s nanoemulsions. Eur. J. Pharm. Sci..

[142] Porter C., Trevaskis N., Charman W. (2007). Lipids and Lipid-Based Formulations: Optimizing the Oral Delivery of Lipophilic Drugs. Nature Reviews Drug Discovery.

[143] Trevaskis N.L., Charman W.N., Porter C.J.H. (2008). Lipid-based delivery systems and intestinal lymphatic drug transport: A mechanistic update. Adv. Drug Deliv. Rev..

[144] Cornaire G., Woodley J., Hermann P., Cloarec A., Arellano C., Houin G. (2004). Impact of excipients on the absorption of P-glycoprotein substrates in vitro and in vivo. Int. J. Pharm..

[145] Zaki N.M. (2016). Strategies for oral delivery and mitochondrial targeting of CoQ10. Drug Deliv..

[146] Read J.L., Whittaker R.G., Miller N., Clark S., Taylor R., McFarland R., Turnbull D. (2012). Prevalence and severity of voice and swallowing difficulties in mitochondrial disease. Int. J. Lang. Commun. Disord..

[147] Re G.L., Terranova M.C., Vernuccio F., Calafiore C., Picone D., Tudisca C., Salerno S., Lagalla R. (2018). Swallowing impairment in neurologic disorders: The role of videofluorographic swallowing study. Pol. J. Radiol..

[148] Masotta N.E., Martinefski M.R., Lucangioli S., Rojas A.M., Tripodi V.P. (2019). High-dose coenzyme Q10-loaded oleogels for oral therapeutic supplementation. Int. J. Pharm..

[149] You Y.-Q.N., Ling P.-R., Qu J.Z., Bistrian B.R. (2008). Effects of medium-chain triglycerides, long-chain triglycerides, or 2-monododecanoin on fatty acid composition in the portal vein, intestinal lymph, and systemic circulation in rats. JPEN J. Parenter. Enter. Nutr..

[150] Ashwell M. (2002). Concepts of Functional Foods.

[151] Yang H., Song J. (2006). [Inclusion of coenzyme Q10 with beta-cyclodextrin studied by polarography]. Yao Xue Xue Bao.

[152] Žmitek J., Šmidovnik A., Fir M., Prošek M., Žmitek K., Walczak J., Pravst I. (2008). Relative bioavailability of two forms of a novel water-soluble coenzyme Q10. Ann. Nutr. Metab..

[153] Wang Y., Hekimi S. (2020). Micellization of coenzyme Q by the fungicide caspofungin allows for safe intravenous administration to reach extreme supraphysiological concentrations. Redox. Biol..

[154] Pepić I., Lovrić J., Hafner A., Filipović-Grčić J. (2014). Powder form and stability of Pluronic mixed micelle dispersions for drug delivery applications. Drug Dev. Ind. Pharm..

[155] Rabanel J.-M., Aoun V., Elkin I., Mokhtar M., Hildgen P. (2012). Drug-Loaded Nanocarriers: Passive Targeting and Crossing of Biological Barriers. Curr. Med. Chem..

[156] Rangel-Yagui C.O., Pessoa A., Tavares L.C. (2005). Micellar solubilization of drugs. J. Pharm. Pharm. Sci..

[157] Nir Y., Paz A., Sabo E., Potasman I. (2003). Fear of injections in young adults: Prevalence and associations. Am. J. Trop. Med. Hyg..

